# Systemic Mechanisms of Ionic Regulation in Carcinogenesis

**DOI:** 10.3390/cancers17020286

**Published:** 2025-01-17

**Authors:** Tatiana N. Zamay, Sergey S. Zamay, Galina S. Zamay, Olga S. Kolovskaya, Anna S. Kichkailo, Maxim V. Berezovski

**Affiliations:** 1 Federal Research Center “Krasnoyarsk Science Center” of the Siberian Branch of the Russian Academy of Sciences, Laboratory for Digital Controlled Drugs and Theranostics, Molecular Electronics Department, 660036 Krasnoyarsk, Russia; sergey-zamay@yandex.ru (S.S.Z.); galina.zamay@gmail.com (G.S.Z.); olga.kolovskaya@gmail.com (O.S.K.); annazamay@yandex.ru (A.S.K.); 2 Prof. V.F. Voino-Yasenetsky Krasnoyarsk State Medical University Laboratory for Biomolecular and Medical Technologies, 660022 Krasnoyarsk, Russia; 3 Department of Chemistry and Biomolecular Sciences, University of Ottawa, Ottawa, ON K1N 6N5, Canada; maxim.berezovski@uottawa.ca

**Keywords:** ion homeostasis, carcinogenesis, sodium, potassium, calcium, cell volume, proliferation, proliferative pathologies, functional system, cancer biomarkers

## Abstract

Cancer is a complex disease characterized by uncontrolled cell proliferation at various levels, leading to tumor growth and spread. This review focuses on the role of ion homeostasis in cancer progression. It describes a model of ion-mediated regulation in both normal and cancerous cell proliferation. The main function of this system is to maintain the optimal number of cells in the body by regulating intra- and extracellular ion content. The review discusses the key points of ion regulation and their impact on tumor growth and spread during cancer development. It explains that normal levels of sodium, potassium, calcium, chloride, and hydrogen ions are regulated at different levels. Damage to ion transport mechanisms during carcinogenesis can lead to an increase in sodium cations and water content in cells, disrupting the balance of calcium and hydrogen ions. This, in turn, can lead to chromatin compaction reduction, gene overexpression, and instability at the epigenetic and genomic levels, resulting in increased cell proliferation and mutagenesis. Restoring normal ion balance can reduce the proliferative potential of both normal and tumor cell populations. The proposed model of systemic ionic regulation of proliferation aims to reconcile diverse data related to cell mitotic activity in various physiological conditions and explain tumor growth. Understanding the mechanisms behind pathological cell proliferation is important for developing new approaches to control ion homeostasis in the body, potentially leading to more effective cancer treatment and prevention.

## Simple Summary

Cancer occurs when cells grow uncontrollably, leading to the formation of tumors. This review examines how maintaining a balance of essential minerals, known as ions, in our cells influences the progression of cancer. It explains that normal levels of ions like sodium and calcium are necessary for healthy cell function. When ion levels are disrupted during cancer, it can lead to rapid cell growth and changes in genes. By restoring the right balance of these ions, we might be able to slow down the growth of both cancerous and normal cells, which could help improve cancer treatments and prevention methods.

## 1. Background

Cancer is a systemic disease that occurs due to a combination of regulatory disorders in cell proliferation at the gene, cellular, and organismic (systemic) levels. Normally, the proportion of proliferating cells in the organs, which determines their size, is regulated at the level of the body using negative feedback. This proportion reflects its functional needs; it is determined by the genetic program of metabolism, differentiation, and specialization of the organ and depends on the availability of metabolic substrates and the influence of external factors [1,2,3].

**Figure 1:**
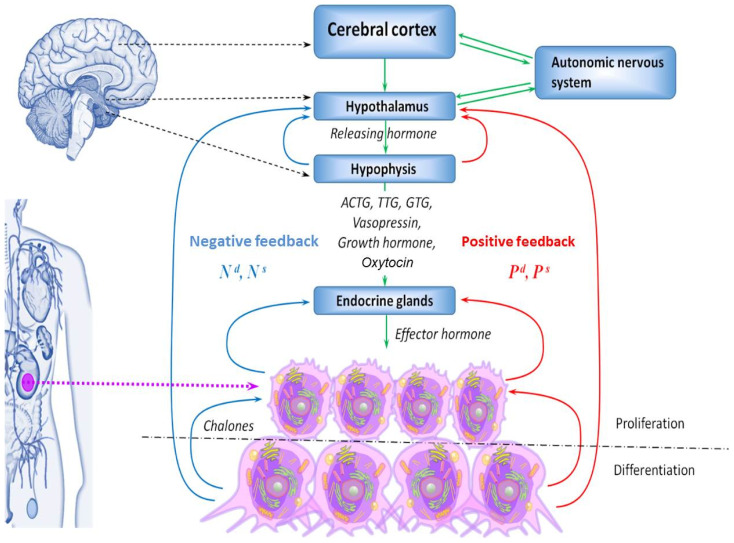
Multilevel regulation of proliferation. Nd, Ns (regulation by deviation)–signals that ensure the regulation of cell proliferation and differentiation in tissues through negative feedback mechanisms, leading to the restoration of homeostatic parameters. Nd is the signal that reduces the effect of the activating stimulus when the required level of differentiated cells in the organ is restored, which is sufficient for this organ to perform its essential function for the organism; Ns is the signal that stimulates the proliferation and differentiation of cells in the organ in the case of functional insufficiency. Pd, Ps (regulation by perturbation)–signals that provide regulation of the system through positive feedback mechanisms, driving it to a new level of homeostasis. Pd is the signal that stops the effect of the activating stimulus when functional sufficiency of the organ is achieved while functioning under new conditions at a new level; Ps is the signal that continues the action of the activating stimulus in the event that functional sufficiency of the organ is not achieved while functioning under new conditions. Thus, positive and negative feedback mechanisms regulate the balance between proliferating and differentiating cells in tissues [2]. In carcinogenesis, for various reasons, an excessively active signal Ps leads to persistent stimulation of cell proliferation. The blue and red arrows indicate negative and positive feedback, respectively. The green arrow represents a local autonomic influence. The purple arrows schematically illustrate cell enlargement in the organ.

It is especially worth noting the role of mitochondria, the functional activity of which is determined by various metabolites and inorganic ions [4], and, in turn, they are involved in the regulation of cell growth [5]. All multicellular organisms have a unique system of hierarchically organized mechanisms for regulating cell proliferation in tissues and organs, providing a balance between growth-stimulating and growth-inhibiting signals, which allows for controlling the organ’s size according to the body’s functional needs. Each cell in the composition of the population performs its characteristic functions and represents a complex, functional self-regulating system. All its components are interconnected, and signaling systems normally allow performing cellular functions at a level adequate to the needs of the body at a particular point in time (Figure 1).

**Figure 2:**
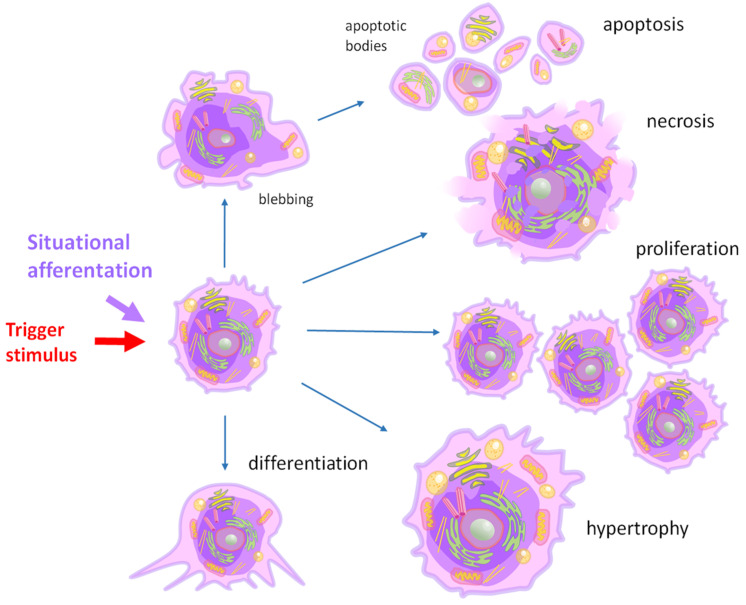
Changes in cell functional state under external stimuli. Depending on the duration of exposure and the strength of the stimulus, cells activate processes of hypertrophy and proliferation to maintain their functional state. A prolonged stimulus capable of causing functional and/or morphological disturbances in a cell can stimulate uncontrolled proliferation. If the stimulus acting on the cell is incompatible with its survival, the cell initiates a process of apoptosis or necrosis. In a fully formed adult organism under stationary conditions, the number of cells in the organ remains generally unchanged. The stability of cell populations is maintained by the balance between proliferating, dying, and differentiated cells. This balance is regulated by central and peripheral mechanisms using the negative feedback system, which is performed by chalones produced by differentiated cells and acting on proliferating cells (Figure 1). Chalones are tissue hormones. They are tissue-specific and act in the late G1 phase of the cell cycle, preventing DNA synthesis, or in the late G2 phase, controlling mitotic activity. The more differentiated cells in the tissue, the higher the level of chalones and the lower the rate of cell proliferation. The concentration of chalones is directly proportional to the number of cells in the organ. When the organ reaches a certain size, the concentration of the chalone is sufficient to stop the growth [10]. For example, skeletal muscle and myocardial growth are negatively regulated by myostatin, the concentration of which depends on the muscle mass itself [2]. Such growth-inhibitory signals can act locally or circulate and act systemically.

The need of the body to increase the functional activity of the organ can cause hypertrophy of its individual cells [6,7] or their hyperproliferation [8] (Figure 2). Under conditions of embryonic and postembryonic development, adaptation to stress factors, and regeneration, the balance between growth-stimulating and growth-inhibiting signals is temporarily shifted towards growth-stimulating ones. With oncogenic transformation, this shift becomes almost irreversible. In normal and neoplastic cells, stimulation of proliferation occurs due to the activation of the expression of genes that control the cell cycle [9].

Normally, the proportion of cells at rest in the tissues is significant. Each tissue, depending on the operating conditions, is characterized by its own unique value of the proliferative pool of cells. In addition, there is a constant change in the periods of rest and activity in the tissues, which is necessary to coordinate the growth of the organ and regulate the number of cells in it. This alternation of periods of active division and proliferative rest gives flexibility and, at the same time, stability to the entire regulatory system. In carcinogenesis, the alternation of periods of rest and proliferation is disrupted, and there is an increase in the number of proliferating cells.

There is no single point of view on the mechanisms of interaction between different levels of regulation of proliferation, both genomic, cellular, tissue, organ, and organism, without which it is difficult to trace a causal relationship and, thus, understand the etiology of carcinogenesis. It is natural to assume that the main connecting element must be sought among signaling systems that permeate all levels of regulation of proliferation. Such systems should be primarily aimed at maintaining the parameters of the body’s homeostasis. The main prerequisite for determining such a binding signaling system is studies showing the dependence of cell proliferative activity on inorganic ion balance. Based on this fact, it can be assumed that inorganic ions (K^+^, Na^+^, Ca^2+^, Cl^−^, HCO_3_^−^, H_3_O^+^, Mg^2+^, Zn^2+^, Fe^2+^/Fe^3+^ and others) are those binding elements that combine the genomic, cellular, and organismic levels of regulation of cell proliferation. However, to fully understand the role of inorganic ions in the systemic regulation of proliferation, it is necessary to consider the relationship between the balance of ions and the cellular parameters that depend on it with the central mechanisms of proliferation regulation.

## 2. Ion Balance During Carcinogenesis

**Figure 3:**
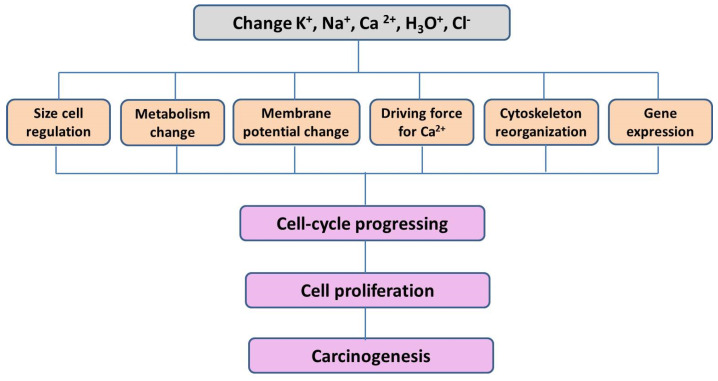
The key role of inorganic ions in the functional state of the cell under normal conditions and during carcinogenesis.

Ions are suitable candidates for linking the genomic, cellular, and organismic levels of regulation of proliferation, since they respond to any external signals. Ions are able to respond to all, even weak, extracellular stimuli by changing their concentrations in the cytosol; they have effective mechanisms to maintain their balance and have the ability to quickly and reversibly change the conformation of protein molecules [11]. In addition, they regulate the main metabolic pathways in the cell, help maintain membrane potential, and regulate proliferation [12,13]. Inorganic ions (K^+^, Na^+^, Ca^2+^, Cl^−^, HCO^3−^, and H_3_O^+^) are the key ions involved in the primary response of the cell to any effects in normal and pathological conditions [14,15,16,17,18,19,20,21,22,23,24,25,26]. The following parameters regulate cell size, metabolism level, membrane potential, intracellular calcium level, and gene expression (Figure 3).

A change in the functional state of the cell is accompanied by a shift in the balance of the main ions. For example, in dividing cells, the sodium content is higher than in dormant cells. However, the sodium content of dividing normal cells is less than that of tumor cells [24,27,28]. Excessive intracellular sodium and low intracellular potassium predispose to malignant mitogenesis [28].

Another important ion that regulates the cell cycle is a hydrogen ion [29,30]. The increase in pH in tumor cells can be stimulated by various factors, but activation of the Na^+^/H^+^-exchanger of the plasma membrane, which removes H^+^ from the cell, is considered to be the main mechanism for increasing the pH [31,32]. The implantation of a cell line with mutations of the Na^+^/H^+^-exchanger in immunodeficient mice causes tumor growth. Stimulation of Na^+^/H^+^ exchange always leads to alkalization, and, as a result, the concentration of growth-inducing factors in resting cells increases [33]. The growth factors on which cell division depends are less active in cells with a deficiency of Na^+^/H^+^-exchanger [34] and in cells in which it is pharmacologically inhibited [35], as well as in cells with mutant NHE1, which is not capable of translocation of protons. Alkalization of the cytoplasm [36], acidification of the culture medium, or fixation of pH in not yet transformed cells leads to an increase in the rate of division. The mechanism providing intracellular alkalization is a key event in oncogenic transformation necessary for the development and maintenance of the transformed phenotype [36,37]. A decrease in the concentration of intracellular sodium significantly inhibits proliferation [36].

## 3. Cell Membrane Depolarization During Carcinogenesis

**Figure 4:**
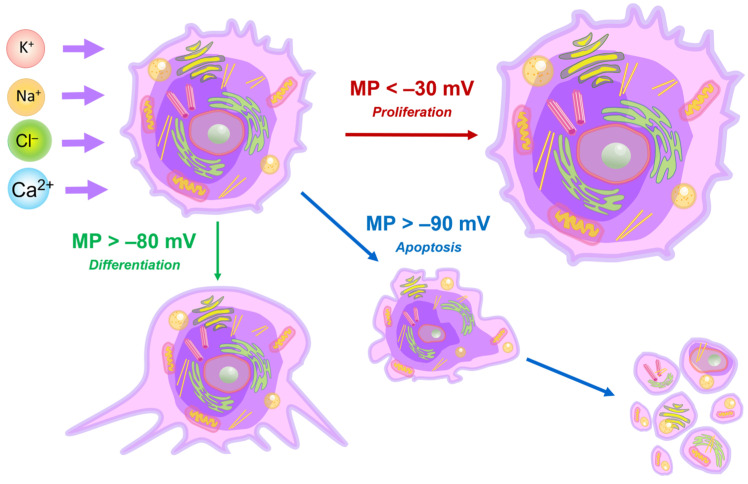
Ion imbalance causes changes in cellular membrane potential (MP) and cell size induces cell differentiation, proliferation, or apoptosis. A membrane potential of more than −80 mV stimulates the process of cell differentiation, while a potential of more than −90 mV induces the transition of the cell into a state of apoptosis. Additionally, cell depolarization stimulates its proliferation. The red, blue, and green arrows indicate necrosis, apoptosis, and proliferation, respectively.

The imbalance of ions in a tumor cell leads to the depolarization of its membrane, an increase in cell volume, and proliferation induction. Hyperpolarization causes cell size change and differentiation or apoptosis (Figure 4). The average values of the membrane potential (MP) in tumor cells during the course of the cell cycle are also lower than in non-tumor cells undergoing the cell cycle. The low membrane potential of tumor cells causes the reorganization of plasma lipids and the activation of membrane-related signaling pathways that stimulate the entry of calcium cations into cells [38]. An increase in the concentration of calcium ions in the cell has many effects, including facilitating the clustering of the K-Ras protein bound to the cell membrane due to electrostatic interactions with phosphatidylserine and enhancing signaling of the K-Ras-dependent mitogen-activated protein kinase (MAPK), which plays an important role in key cellular processes, including proliferation. Conversely, plasma membrane hyperpolarization disrupts K-Ras clustering and inhibits MAPK signaling [38].

Thus, ion flows change the potential of the cell membrane, regulate the proliferation process [12,13,39,40,41], and control the regeneration, morphogenesis, differentiation, and migration of cells [23,42]. The differentiation of cells is stimulated by a ratio of ions that causes hyperpolarization of the membrane [43], while a decrease in membrane potential prevents the differentiation of cells [44,45]. The depolarization of membranes makes cells more immature, transferring them to the level of stem cells. Cells with high proliferative potential have a lower membrane potential compared to differentiated cells [46] and enhance the expression of various passive channels [47] and active electrogenic transporters [48], participate in cell communication [49], and regulate mitosis, regeneration, morphogenesis, gene expression, cell growth, apoptosis, and left-right asymmetry. An artificial change in membrane potential causes a change in morphogenesis [50]. The depolarization of the membrane of a cancer cell due to the accumulation of excessive negative ions inside the cell stimulates the processes of carcinogenesis. It has been established that membrane depolarization induces mitotic activity through the activation of extracellular signal-regulated kinase (ERK) [51].

All known ion channels participate in the generation of membrane potential [52,53,54,55,56,57]. Ion channels support the malignant behavior of tumor cells [52,53,56,57]. A pharmacological blockade of ion channels that increases membrane potential can inhibit the proliferation and growth of cancer cells [56,58]. It is assumed that the normalization of membrane potential in cells of the body can become a key component of cell restoration and cancer treatment.

## 4. Changes in Cell Volume During Carcinogenesis 

**Figure 5:**
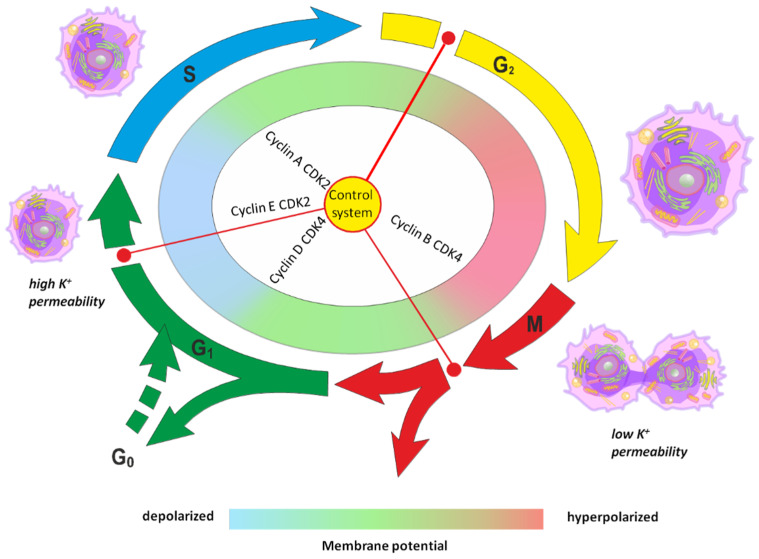
Change in potassium permeability, membrane potential, and cell volume during the cell cycle. The G1-to-S phase transition in the cell cycle is accompanied by membrane hyperpolarization due to potassium cation efflux. Conversely, prior to the M phase transition, decreased potassium cation permeability leads to membrane depolarization and initiates cell division. Quiescent G0 phase cells only stimulate mitotic activity following membrane depolarization. Violations of the regulation of cell volume, regardless of the cause, contribute to the development of many diseases, including cancer. A change in cell volume caused by a violation of intracellular homeostasis of sodium, potassium, and calcium cations, when it is impossible to be compensated by adaptive mechanisms, causes many pathologies [66].

The volume of the cell is one of the system-forming parameters of the cell’s life, the constancy of which the cell seeks to provide with mechanisms of molecular-cellular regulation of ionic homeostasis. To maintain their volume in a stable state, cells use many mechanisms, both transport and metabolic. Moreover, hormones and neurotransmitters can modify cellular size [59]. Proliferation processes are accompanied by an increase in cell volume [60,61,62,63] and membrane depolarization [25]. The increase in cell volume occurs in parallel with the transition of cells from stage G1 to stage S and is accompanied by the inhibition of K^+^-channels, the activation of Na^+^/H^+^-exchange, and Na^+^-K^+^-2Cl^−^-cotransport. Normal cell cycle progression of the multicellular organism is of vital importance because it defines body size and shape, tissue regeneration, and aging. The cell cycle is a complex process with multiple regulation mechanisms. During the resting phase, normal cells maintain a high membrane potential ranging from −60 to −100 mV. However, upon initiation of cell division and DNA synthesis, the membrane potential decreases to approximately −15 mV [64,65]. Persistent elevation of intracellular sodium levels can act as a mitotic trigger, compelling cells to undergo cell division (mitosis). In a multicellular organism, the progression of the cell cycle is crucial for maintaining normal body size and shape, tissue renewal, and aging. The cell cycle is a complex process with numerous regulatory mechanisms (Figure 5).

The ratio of sodium, potassium, and calcium cations, which determines the volume of the cell, is the basis of the fundamental mechanism for regulating proliferation in both normal and tumor cells. The content of potassium and sodium cations in the tissues of an early cancerous tumor compared to normal tissue is doubled, while the water content is increased. A correlation was established between proliferative activity and an increase in the content of sodium cations in tumor and normal cells with different mitogenic activity. Thus, it is obvious that tissue malignancy is associated with a violation of ionic homeostasis, which in turn stimulates mitotic activity. One of the most probable reasons for the influence of cell volume on its functional state is the dependence of the biological function of proteins on their conformation. The conformation of a protein molecule, and, consequently, its biological activity, directly depends on its hydration [67,68].

The calculations showed that, on average, there are about 10^5^ water molecules per protein molecule, and each amino group in a protein molecule binds on average 2.6 water molecules. The ratio of the number of hydrogen bonds between adjacent turns of the helix and the number of hydrogen bonds between turns through water molecules determines the length of a particular protein molecule under specific conditions. Consequently, increased hydration of the protein molecule causes elongation of the molecule, and weakened hydration leads to a reduction in its length. The more bonds a protein molecule has with water molecules, the more it is stretched. The more hydrogen bonds, the stronger the hydration and the greater the magnitude of the forces stretching the protein molecule. The forces of covalent bonds, forming a protein molecule, oppose these forces. The length of a protein molecule at any given time depends on the ratio of these forces [11]. A change in the concentration of positively hydrated ions in the cell, in particular sodium cations, changes the hydration and, consequently, the state of protein molecules and their biological function [67,68,69]. In a normal cell, proteins are primarily associated with potassium rather than sodium [70]. Based on experimental data, Cope F.W. definitively concluded that, when a cell is damaged, it typically swells, and the content of unbound water within it increases, resulting in a disruption of protein conformation and a decrease in its binding to potassium. When the intracellular water content decreases, the conformation of protein molecules is restored [70].

Furthermore, protein conformation, and, consequently, biological function, is dependent on protonation and the pH level [71,72]. Protein protonation alters protein–protein interactions [73] and ligand binding [72]. Therefore, protonation represents a readily available and evolutionarily conserved mechanism for regulating protein biological activity, serving as a form of post-translational modification. By altering the charge of amino acid side chains, post-translational modification via protonation can induce dynamic changes in protein conformation and function. Proton addition and removal are rapid and reversible and, unlike most other post-translational modifications, do not require enzymatic catalysis.

Therefore, changes in cell volume and alkalinization during carcinogenesis modulate the activity of various macromolecules, including proteins involved in cell growth and division signaling pathways, which are determined by their hydration level.

Macromolecules can exist in active hydrated and inactive dehydrated or partially hydrated forms, and a change in water content will change the ratio of active and inactive forms. 

## 5. Disruption of Ion Balance Regulation in the Cell During Carcinogenesis

**Figure 6:**
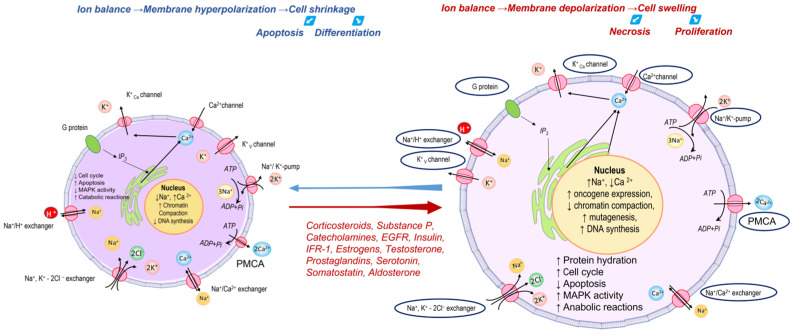
Disruption of ion balance leads to membrane hyperpolarization or depolarization and cell shrinkage or swelling with the following changes in cell states. Ion transporter proteins, ion channels, and ion pumps, whose expression is increased during carcinogenesis, are highlighted [19]. Blue indicates shrinkage, while red indicates swelling.

The maintenance of ionic balance in the cell is carried out through fine coordination of the processes of active and passive transport of cations and anions (Figure 6), the vast majority of which are disrupted during carcinogenesis [19]. The main ion transporters, ion channels, and ion pumps, whose expression increases during carcinogenesis under the influence of steroid hormones, catecholamines, insulin, growth factors, and others, stimulate an increase in cell size. This process is accompanied by an ionic imbalance, which, in turn, leads to the activation of the cell cycle, reduced chromatin compaction, and the induction of mutagenesis (Figure 6). Therefore, it is natural that, regardless of the causal relationship, the increase in sodium cations detected in tumor cells will indicate a steady imbalance of cellular ion-transporting systems. Often, this imbalance is associated with the features of cancer tissue metabolism and is caused by pH regulation mechanisms that protect cells from acidification under conditions of activated glycolysis. The regulation of pH in the cytosol is carried out with the participation of Na^+^/H^+^- and Cl^−^/HCO_3_^−^-exchangers, Na^+^/HCO_3_^−^-cotransporter, and Na^+^-independent HCO_3_^−^/Cl^−^-antiporter. Moreover, the Na^+^/H^+^-exchanger is of particular importance in the regulation of tumor growth and proliferation.

In addition to passive transport, active transport of cations plays an important role in the regulation of proliferation during carcinogenesis. For example, during the induction of experimental cancer in the distal colon of the mouse, inhibition of Na,K-ATPase occurs long before the onset of morphological changes, which leads to an increase in intracellular sodium and a decrease in potassium in precancerous and cancerous cells. Similar changes were found in carcinoma cells, in which the content of sodium cations occurs with a decrease in the activity of Na,K-ATPase. Moreover, the suppression of the Na,K-ATPase activity during carcinogenesis is not associated with a decrease in the level of energy exchange, since the compensatory maintenance of ion homeostasis can be carried out under conditions of reduced energy supply, decreased intracellular ATP, impaired physicochemical properties of the plasma membrane, and ion permeability. A malfunction of the transporter proteins that regulate ionic homeostasis in the cell can be regulatory and occur in response to changes in the content of hormones, cytokines, and neurotransmitters. For example, the insulin-like growth factor IGF-1 activates the electroneutral KCl-cotransporter and, thus, stimulates the proliferation and invasion of tumor cells [74]. Growth factors usually activate Na^+^/H^+^-exchange and stimulate cell swelling, inhibiting proteolysis and inducing proliferation. Therefore, carcinogenesis disrupts the function of most ion channels, leading to its classification as a channelopathy [19].

## 6. Violation of the Systemic Mechanisms of Ionic Regulation of Proliferation During Carcinogenesis

**Figure 7:**
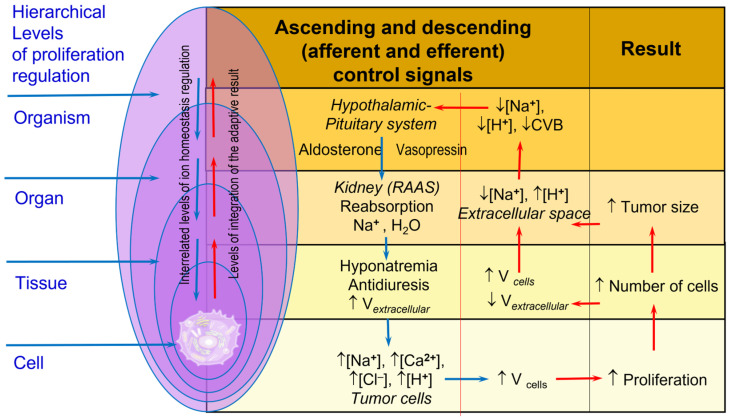
Interaction between different ion balance regulation levels and proliferation in tumor tissues. Elevated intracellular levels of sodium [98], calcium [99], and chloride [100], along with decreased hydrogen ion concentration [101], in tumor cells lead to increased cell volume and stimulate proliferation [23]. This leads to an increased number of tumor cells and a decreased extracellular space volume at the tissue level [98]. The extracellular space shows decreased sodium and increased hydrogen ion concentrations [83,102]. Reduced circulating blood volume [103] stimulates the hypothalamic-pituitary system. Despite compensatory increases in aldosterone and vasopressin, hyponatremia develops. This hyponatremia contributes to excessive tumor cell proliferation [83] due to the resulting intracellular ion imbalance. Red arrows indicate afferent control signal, blue — efferent control signals.

The ionic balance in the human body is maintained with the help of a multilevel system of regulation, the highest center of which is the hypothalamus. The nuclei of the hypothalamus produce releasing factors that regulate the secretion of the hormones of the adenohypophysis: somatotropin, thyrotropin, adenocorticotropin, follitropin, and lutropin. Tropic hormones of the adenohypophysis, in turn, control the secretion of effector hormones involved in maintaining the balance of ions [75,76]. An increase in the concentration of effector hormones (IGF, thyroid hormones, estrogens, testosterone, mineralocorticoids, etc.) in the blood plasma reduces the secretion of releasing factors due to the feedback system. In addition to releasing factors, the hypothalamus produces vasopressin, which, along the axons, enters the neurohypophysis and then into the blood (Figure 7).

Recent evidence suggests that cancer is a systemic disease. Systemic coordination is regulated by common physiological processes, including, but not limited to, cell division. [77]. During carcinogenesis, the hierarchical system of regulation of ionic homeostasis is disrupted, which causes metabolic and proliferative changes in the body. The sympathetic nervous system plays an important role in disturbing the balance of ions, which, during carcinogenesis, activates [78,79] and triggers the hypophyseal portal system involved in tumor progression [80,81]. In addition, vasopressin secretion increases during carcinogenesis, which causes inadequate antidiuresis syndrome [82,83] and hyponatremia [83]. In conditions of hyponatremia, the volume of cells increases, and the cytoskeleton is rebuilt, while cell proliferation, adhesion, and invasion are stimulated [84,85,86]. Chronic hyponatremia stimulates RhoA and HMOX-1 signaling systems that induce microtubule remodeling and oxidative stress, which stimulate tumor growth [87].

In tissues, in addition to the hypophyseal portal system, the diffuse endocrine system, the APUD system (Amine Precursor Uptake and Decarboxylation), is involved in the regulation of ion balance and proliferation. This system secretes biogenic amines and peptide hormones, including the sodium-uretic peptide, insulin, insulin-like growth factor, serotonin, substance P, somatostatin, YY polypeptide, ACTH (adrenocorticotropic hormone), endothelial growth factor, prostaglandins, etc., which can shift the balance of ions and stimulate cell proliferation during carcinogenesis [88,89,90,91,92,93,94]. In addition to the APUD system, the RAAS system (renin–angiotensin–aldosterone) is also present in organs and tissues, which also regulates ion balance and cell proliferation [95,96,97]. Both systems are actively functioning and mutually complementary. The RAAS (renin–angiotensin–aldosterone) system plays an important role in the development of carcinogenesis, as it is responsible for disrupting ion homeostasis, which stimulates cell proliferative activity. Suppression of the RAAS system reduces tumor growth [96,98].

A decrease in the volume of the intercellular space in the tumor tissue, an increase in the concentration of hydrogen ions in the intercellular space [97], and a decrease in the concentration of sodium cations [83] are detected by the sensory mechanisms of the antidiuretic system using peripheral and central osmoreceptors, baroreceptors, and sodium receptors. Using a feedback system, the hypophyseal portal system stimulates the secretion of antidiuretic hormone, which increases the kidneys’ level of water reabsorption and circulating blood volume. An additional regulatory element is the juxtaglomerular apparatus of the kidney, which controls the level of sodium reabsorption through the renin–angiotensin–aldosterone system, which is controlled by the sympathetic nervous system. All regulatory systems taken together cause changes in the balance of sodium, potassium, chlorine, and hydrogen ions in the cells of the tumor tissue, which reduces the potential of the cell membrane and increases the volume of the cell. Thus, the control system of ionic homeostasis of the body regulates the growth of tumor tissue (Figure 7).

## 7. A Model of Ion Regulation of Tumor Growth During Carcinogenesis

Based on a literature analysis, we have developed a model that proposes ion regulation as a key factor in tumor growth during carcinogenesis. The model is based on the following experimental findings:

(1) Cell volume increase stimulates anabolic processes and proliferation.
(2) Cell volume is influenced by the ratio of ions in the intracellular and extracellular space, which in turn determines the membrane potential.
(3) Hormones and biologically active substances that regulate proliferation impact cell volume by modulating the function of ion transporters.

Under normal conditions, the ionic regulation of proliferation is a hierarchical, multilevel system. Its primary function is to maintain a sufficient number of cells in the body, with the level of ions in the extracellular and intracellular space serving as the main controlling parameter of homeostasis. Neuroendocrine mechanisms maintain near-constant values of ion levels, which contribute to the organism’s successful adaptive response as a whole. Cellular mechanisms play a crucial role in maintaining a functional state to ensure the number of cells near a genetically programmed level. These mechanisms correspond to evolutionarily selected and fixed strategies of cell behavior, providing an adaptive response within the entire organism.

Pathological proliferation conditions arise due to the pathological disruption of central mechanisms regulating ionic homeostasis, local disturbances in coordinating mechanisms of ion balance regulation, or the local isolation of cells. If the central regulatory mechanisms lose control over individual elements, temporarily triggered mechanisms for increasing the number of cells may fail to return to the required state, leading to the conditions for carcinogenesis. The degree of carcinogenesis will be determined by the extent of deviations from normal ion balance parameters in the cell population that has become independent of the body.

## 8. Application of the Model to the Design of Anti-Cancer Drugs and the Discovery of Cancer Biomarkers

Ionic homeostasis, due to its multifunctionality, is of critical importance for the viability of living organisms. The significance of mechanisms maintaining ionic homeostasis is confirmed by the fact that sodium ions, through their influence on Na^+^-sensitive riboswitches, regulate the expression of genes involved in key cellular processes. [104,105]. The disruption of sodium homeostasis may be one of the factors inducing genomic instability characteristic of carcinogenesis. At the cellular level, intracellular Na^+^ concentration ([Na^+^]i) regulates pH, [Ca^2+^], membrane potential, metabolism, and proliferation. At the tissue level, high [Na^+^]i promotes malignant cell migration and invasion, while systemic [Na^+^] changes affect blood pressure, immune function, and the secretion of proangiogenic mediators [18]. Therefore, the artificial disruption of ion homeostasis in tumor cells may become a new approach to suppressing their proliferation and, consequently, a promising method for cancer therapy. Traditional drugs typically target only one key pathway, while the conditions that lead to the transformation of a normal cell into a tumor cell are continuously reproduced. Thus, to completely block the malignant process, it is necessary to alter conditions both within the cell and in its microenvironment, i.e., to change the conditions that enable its progression.

Many ion transporters maintain cellular ion balance, with ion channels predominating. Ion channels, a class of proteins involved in virtually all biological processes from cell proliferation to metabolism and cell death regulation, play a crucial role in cancer development [19]. In the last decade, ion channels have been recognized as key factors controlling hallmarks of malignancy [56], leading to the classification of malignancies as channelopathies. Aquaporins (AQPs), voltage-gated calcium channels (Cavs), cystic fibrosis transmembrane conductance regulator (CFTR), chloride intracellular ion channels (CLICs), ATP-sensitive potassium channels (KATPs), voltage-gated potassium channels (Kvs), voltage-gated sodium channels (Navs), store-operated calcium channels (SOCCs), and transient receptor potential channels (TRPs) have been shown to support proliferation signaling. Furthermore, AQPs, Cavs, KATPs, Navs, and TRPs participate in evading growth suppressors; ASICs, calcium release-activated channels (CRACs), calcium-activated potassium channels (KCas), and TRPs are involved in avoiding immune destruction; Cavs and TRPs enable replicative immortality; and ATP-gated P2X receptors (P2Xs) and TRPs promote tumor-associated inflammation. Cavs, CLICs, CRACs, KCas, inward rectifier potassium channels (Kirs), Kvs, the mitochondrial calcium uniporter (MCU), sodium-calcium exchangers (NCXs), TRPs, and voltage-dependent anion channels (VDACs) deregulate cellular metabolism; AQPs, Cavs, CFTRs, CLICs, KATPs, Navs, and TRPs confer resistance to cell death; Cavs, KCas, Kvs, Kirs, and TRPs contribute to genome instability and mutation; AQPs, Cavs, connexins, Kirs, and TRPs promote vasculature; and AQPs, ASICs, calcium-activated chloride channels (CaCCs), Cavs, chloride channels (CLCs), CLICs, connexins, CRACs, KCas, Kirs, two-pore domain potassium channels (K2Ps), Kvs, Navs, P2Xs, and TRPs activate invasion and metastasis [106,107,108,109,110,111,112].

Therefore, ion transporters represent novel therapeutic targets for cancer treatment. Restoring their regulation may improve cellular function, and direct or indirect manipulation of tumor [Na^+^]i, for example, using pharmacological tools, could offer a new treatment option in addition to existing therapies.

Currently, several pharmacological agents with anti-tumor effects are known to normalize intracellular sodium levels by suppressing the activity of ion transporters. For example, the inhibition of pharmacological agents, such as ranolazine, specifically targeting voltage-gated sodium channels (VGSC) that are overexpressed in various human malignancies, inhibits tumor development and metastasis [109,113]. Blocking a Na^+^/K^+^/2Cl^−^ cotransporter (NKCC1) inhibitor weakens hepatocellular carcinoma cell proliferation and invasion in vitro and limits their growth in vivo [110]. Another Na^+^/K^+^/2Cl^−^ cotransporter blocker, furosemide, reduces cell growth by delaying G1-S phase progression in poorly differentiated cells exhibiting high expression and activity of this transporter [114]. Etiliso-propylamiloride, a Na^+^/H^+^ exchange inhibitor, demonstrates anti-tumor effects in non-small cell lung cancer [115].

Several agents restoring normal intracellular sodium cation levels in tumor cells are currently undergoing preclinical and clinical trials [18]. Specifically, the Na^+^/K^+^/2Cl^−^ co-transporter inhibitor bumetanide [110], the sodium-calcium exchanger (NCX) inhibitor KB-R7943 [116], the Na,K-ATPase inhibitor ouabain [117], and the voltage-gated sodium channel (VGSC) blocker ranolazine [118] are in preclinical development. VGSC blockers lidocaine and bupivacaine are already in Phase III trials [119,120].

Given the limitations of traditional chemotherapy—namely, low selectivity and drug resistance—and the key role of inorganic ions in carcinogenesis, a novel anti-cancer strategy has emerged: ion interference therapy. This approach enhances metabolic disturbances in tumor cells and suppresses their proliferation by increasing intracellular levels of bioactive ions [121,122]. Currently, ion interference therapy methods are being developed based on overloading tumor cells with calcium [123], sodium [124], and chloride [125] ions. This induces various types of cell damage, triggering different types of cell death through various mechanisms and ultimately suppressing proliferation and metastasis.

Targeted osmotic lysis (TOL) represents a promising direction in anti-cancer therapy. This novel technology involves the combined stimulation of voltage-gated sodium channels (VGSCs) and pharmacological blockade of Na^+^,K^+^-ATPase, promoting the lysis of highly malignant cancer cells [126,127]. Unlike destructive chemotherapy, TOL lyses only highly malignant cells, which differ from normal cells by their high VGSC expression [126,127].

Thus, blocking ion transporters to normalize ion homeostasis may be a useful adjunct therapeutic strategy for slowing or preventing the development of malignant neoplasms. Alternatively, destroying tumor cells by enhancing their ion imbalance to stimulate lysis may also be a viable anti-cancer approach.

## 9. Conclusions

The key points of the model of ion regulation of tumor growth during carcinogenesis:

1. The concentration of Na^+^, K^+^, Ca^2+^, Cl^−^, and H^+^ in the extracellular and intracellular space is constant and regulated at several hierarchical levels. The concentration of ions in the extracellular and intracellular space is disturbed during carcinogenesis.
2. The ratio of cations in the extracellular and intracellular space determines osmolarity and cell volume. The volume of the cell determines its functional state, in particular, its ability to undergo the cell cycle. An increase in cell volume activates anabolic reactions and stimulates the cell to undergo a cell cycle. A decrease in cell volume activates catabolic reactions and apoptosis. Cell volume increases during carcinogenesis.
3. An increase in cell volume activates the expression of genes, including oncogenes, and stimulates mitogen-activated protein kinases. One of the reasons for increased gene expression is a decrease in the concentration of calcium cations in the nucleus, which reduces chromatin compaction, thereby accelerating the passage of the cell cycle and increasing the likelihood of epigenetic and genomic instability that stimulates carcinogenesis.
4. Enhancing anaerobic energy production processes in a tumor cell increases its hydrogen proton concentration, which stimulates Na^+^/H^+^ exchange and promotes the acidification of the extracellular space and alkalization of the intracellular space.
5. The sympathetic nervous system, activated during carcinogenesis by acting on the hypophyseal portal system, APUD system, and the renin-angiotensin system, constantly shifts the balance of ions. Stimulation of the central mechanisms of ionic homeostasis regulation continues until deviations of the H^+^ and Na^+^ levels are observed in the intercellular medium. Deviations in the level of ions causing an increase in cell volume are accompanied by the activation of proliferative processes.
6. Damage to the mechanisms of ion transport and the cell’s inability to maintain the intracellular balance of ions leads to an increase in the concentration of sodium cations and water in the cells and a violation of the balance of calcium cations and hydrogen protons. This reduces chromatin compaction, stimulates gene overexpression, increases the level of epigenetic and genomic instability, and, as a result, hyperproliferation and mutagenesis.
7. A tumor cell is a striking example of damage to the mechanisms of maintaining ionic homeostasis. It has an abnormally high sodium cation concentration, an increased volume, and, therefore, a high proliferative potential.
8. Restoring a normal level of ion balance reduces the proliferative potential of both normal and tumor cell populations.

Cancer is a systemic disease arising from a complex process failure, inadequately regulated at a higher level or malfunctioning at an intermediate level. The proposed model of the systemic mechanisms of ionic regulation of proliferation allows for combining disparate data related to the regulation of mitotic activity of cells in various physiological conditions and explaining tumor growth, which opens up avenues for controlling these processes in the body.
